# Genotyping assay for differentiation of wild-type and vaccine viruses in subjects immunized with live attenuated influenza vaccine

**DOI:** 10.1371/journal.pone.0180497

**Published:** 2017-07-07

**Authors:** Victoria Matyushenko, Irina Isakova-Sivak, Tatiana Smolonogina, Irina Dubrovina, Tatiana Tretiak, Larisa Rudenko

**Affiliations:** Department of virology, Institute of Experimental Medicine, Saint Petersburg, Russia; Georgia State University, UNITED STATES

## Abstract

Live attenuated influenza vaccines (LAIVs) are considered as safe and effective tool to control influenza in different age groups, especially in young children. An important part of the LAIV safety evaluation is the detection of vaccine virus replication in the nasopharynx of the vaccinees, with special attention to a potential virus transmission to the unvaccinated close contacts. Conducting LAIV clinical trials in some geographical regions with year-round circulation of influenza viruses warrants the development of robust and reliable tools for differentiating vaccine viruses from wild-type influenza viruses in nasal pharyngeal wash (NPW) specimens of vaccinated subjects. Here we report the development of genotyping assay for the detection of wild-type and vaccine-type influenza virus genes in NPW specimens of young children immunized with Russian-backbone seasonal trivalent LAIV using Sanger sequencing from newly designed universal primers. The new primer set allowed amplification and sequencing of short fragments of viral genes in NPW specimens and appeared to be more sensitive than conventional real-time RT-PCR protocols routinely used for the detection and typing/subtyping of influenza virus in humans. Furthermore, the new assay is capable of defining the origin of wild-type influenza virus through BLAST search with the generated sequences of viral genes fragments.

## Introduction

Although influenza is a vaccine preventable disease, severe influenza is still a major cause of early childhood deaths in low-income countries [1], mostly due to the unavailability of cheap, safe and effective influenza vaccines in these countries. Recently a study evaluating safety and immunogenicity of a trivalent seasonal Russian-backbone live attenuated influenza vaccine (LAIV) in children 2 to 5 years of age was conducted in Bangladesh. The vaccine was proved to be safe for this age group without evidence of increased frequencies of adverse events in a vaccine cohort compared to a placebo group [2]. An important part of the LAIV safety evaluation is the detection of vaccine virus replication in the nasopharynx of the vaccinees, with special attention to a potential virus transmission to the unvaccinated close contacts. As was demonstrated for another cold-adapted LAIV based on Ann Arbor backbone, the vaccine virus shedding was the highest among young children, and was decreased with increasing age [3–5]. In addition, a single confirmed case of vaccine transmission to an unvaccinated child was reported in one of the studies [4].

The detection of vaccine virus in these studies was based on the culturing nasal swab specimens in susceptible cell culture followed by a genotyping analysis of all positive samples to confirm the presence of specific LAIV component [3]. Since all the studies were conducted outside the influenza season, any sample determined to be influenza-positive by culture were assumed to be vaccine-type virus regardless of whether vaccine genotype could be confirmed [3]. This assumption relies on the distinct patterns of yearly influenza outbreaks in temperate climate regions in both Northern and Southern Hemispheres [6]. In contrast to the temperate regions, there are more complicated patterns of influenza activity in the tropics and subtropics, with year-round circulation in some regions and bi-annual peaks of circulation in others [7]. While assessing LAIV virus shedding in vaccinated subjects in the tropical regions, one need to keep in mind that some of the influenza-positive specimens might arise from natural infection, rather than vaccination. Therefore, there was an urgent need for reliable and sensitive assay to differentiate wild-type influenza virus from vaccine-type virus, for both type A and type B components of trivalent LAIV. Here we report the development of a genotyping assay for the detection of wild-type and vaccine-type influenza virus genes using Sanger sequencing of their short regions from newly designed universal primers.

## Materials and methods

### Ethics statement

Embryonated chicken eggs are routinely used for virus isolation and cultivation. Chicken embryos were purchased from «Nazia» Chicken farm (Kirovsk Area, Leningrad region, Russia). The handling of chicken embryos was performed in accordance with the “Manual for laboratory animals and alternative models in biomedical research” [8]. Ten to twelve days old fertilized eggs were inoculate with influenza virus and incubated for up to three days at 33°C and then chilled at 4°C overnight. After harvest of allantoic fluid the eggs were appropriately discarded according to Russian Sanitary-epidemiological rules SP 1.3.2322–08 (approved 28 Jan 2008). Nasal pharyngeal wash (NPW) specimens tested in this study were obtained from two clinical trials: NCT01625689 and NCT01797029. The details of the study design, protocols and outcomes of the trials can be found elsewhere [2,9]. In the event of inadvertent hatching the chicks were to be euthanized with an overdose of ether. However, no accidental hatches occurred in the course of this study.

### Viruses and clinical specimens

A number of wild-type influenza A and B viruses, as well as cold-adapted master donor viruses were used in this study (Table 1). The viruses were amplified in embryonated chicken eggs at optimal temperature 33°C for 48 hours (for influenza A) and 72 hours (for influenza B), clarified by low-speed centrifugation and stored at -70°C until RNA extraction. In addition, a vial of Russian-backbone trivalent LAIV produced by Serum Institute of India (SII) for influenza season 2012–2013 was used in the study. The vaccine was reconstituted in 0.5 ml of sterile distilled water prior to viral RNA extraction.

**Table 1:** List of influenza viruses used in the study.

Type	Subtype/lineage	Strain name	Origin	wt/ca
A	H1N1	A/California/07/2009	human	wt
	H2N2	A/Leningrad/134/17/57	human	ca
	H2N2	A/California/1/66	human	wt
	H3N2	A/Brisbane/10/2007	human	wt
	H5N2	A/duck/Potsdam/1402-6/1986	avian	wt
	H6N1	A/herring gull/Sarma/51C/2006	avian	wt
	H7N3	A/mallard/Netherlands/12/2000	avian	wt
	H7N9	A/Anhui/1/2013	human^†^	wt
	H9N2	A/Hong Kong/1073/99	human^†^	wt
	H11N9	A/duck/Moscow/3641/2008	avian	wt
B	n/a	B/USSR/60/69	human	ca
	Victoria	B/Nevada/1/2011	human	wt
	Yamagata	B/Texas/26/2008	human	wt

^†^avian influenza virus isolated from infected human subject

n/a, not applicable

Influenza-positive nasal pharyngeal wash (NPW) specimens were provided by International Centre for Diarrheal Disease Research Bangladesh, Dhaka, Bangladesh (ICDDR,B) from two consecutive clinical trials of Russian-backbone trivalent seasonal LAIV in children 24–59 months of age: a phase II safety and immunogenicity study conducted in 2012, and a phase III efficacy study conducted in 2013 [2]. Totally 356 masked NPW specimens were analyzed, 287 of them from the phase II study, and 69 from the phase III trial.

SII-produced trivalent LAIV used in the phase II trial in Bangladesh contained the following reassortant viruses:

- A/17/California/2009/38 (H1N1), a 6:2 reassortant possessing HA and NA genes of A/California/7/2009 (H1N1) and six remaining genes of A/Leningrad/134/17/57 (Len/17) master donor virus;
- A/17/Perth/09/87 (H3N2), a 6:2 reassortant possessing HA and NA genes of A/Perth/16/2009 (H3N2) on Len/17 backbone;
- B/56/Brisbane/60/08, a 6:2 reassortant possessing HA and NA genes of B/Brisbane/60/08 (Victoria lineage) on B/USSR/60/69 backbone.

SII-produced trivalent LAIV used in the phase III trial in Bangladesh contained the following reassortant viruses:

- A/17/California/2009/38 (H1N1);
- A/17/Victoria/2011/89 (H3N2), a 6:2 reassortant possessing HA and NA genes of A/Victoria/361/2011 (H3N2) on Len/17 backbone;
- B/60/Wisconsin/2010/125, a 6:2 reassortant possessing HA and NA genes of B/Wisconsin/1/2010 (Yamagata lineage) on B/USSR/60/69 backbone.

The NPW specimens were shipped from the study center to the Institute of Experimental Medicine on dry ice and then stored at -70°C until use.

### Design of universal primers

For the purpose of designing universal primers within all eight genes of influenza A and B viruses we looked for highly conserved regions inside these genes where a primer with desired properties (molecular weight, melting temperature, the lack of self-annealing and hairpin formation etc.) could be annealed. To do so we selected 29 influenza A(H1N1), 29 influenza A(H3N2) and 34 influenza B (from both Victoria and Yamagata lineages) evolutionary diverged strains of various years of isolation and compared nucleotide sequences of their genes with those of master donor viruses for LAIV: A/Leningrad/134/17/57 (H2N2) and B/USSR/60/69. We used Influenza Research Database (IRD) (http://www.fludb.org/) to select representative strains from evolutionary diverged lineages through generation of phylogenetic trees from over 1500 viruses. Then the selected sequences were analyzed by Multiple Sequence Alignment tool of Influenza Research Database to identify conserved regions where a primer can be generated. In some cases universal primers designed by Hoffmann et al. were used in pairs with the newly designed ones [10,11].

Analysis of non-specific annealing of the universal primers to human DNA was performed using on-line resource Primer Blast (https://www.ncbi.nlm.nih.gov/tools/primer-blast), with Genome Reference Consortium Human Build 38 patch release 7 (GRCh38.p7) as a template, GenBank assembly accession: GCA_000001405.22 (replaced). Search was done with primer optimal annealing temperature 55°C, mismatch allowance of 5 nucleotides and PCR product length of less than 2000 bp.

### RNA extraction, RT-PCR and Sanger sequencing

Viral RNA was extracted from the allantoic fluid or NPW specimen using QIAamp Viral RNA Mini Kit (QIAGEN) according to the manufacturer’s instructions. RT-PCR was performed using SuperScript III One-Step RT-PCR System with Platinum Taq DNA Polymerase (Invitrogen). The following reaction conditions were used: the PCR mixtures contained 12.5 μl of 2× mix, 1 μl of enzyme mixture SuperScript III, 0.7 μl of 20 pM of forward and reverse primers, 2.6 μl of viral RNA and 7.5 μl of distilled RNA-, DNAase free water (QIAGEN). The first and second cycles of the amplification program consisted of a one 30-min period at 60°C and one 2-min period at 94°C were followed by 40 cycles with the following conditions: 94°C for 15 sec, 55°C for 30 sec and 68°C for 1 min. The program ended with one cycle at 68°C for 5 min.

The RT-PCR products were separated by electrophoresis on 0.9% agarose gels and the excised DNA bands were purified by Omnix Kit for Extracting DNA from PCR mixture and agarose gel (Andromed, Russia) according to the manufacturer’s protocol, with the exception that the amplified DNA fragment was eluted in 12 μl of the elution buffer. The DNA fragments were sequenced with the BigDye Terminator v3.1 Cycle Sequencing Kit (Applied Biosystems) using either of the universal primers used for RT-PCR. The nucleotide sequences were generated with an ABI 3130xl Genetic Analyzer (Applied Biosystems). The resulted sequences were analyzed with DNAStar Lasergene software v7.1, and then compared to the known sequence of the master donor viruses. In case the sequences didn’t match, a BLAST search with the newly obtained sequence was performed to find the closest wild-type influenza virus. The generated partial sequences of wild-type viral genes were deposited into GISAID repository (see accession numbers in S1 Table). Evolutionary relationship of the identified H1N1pdm viruses was demonstrated using the Archaeopteryx Phylogenetic Tree Viewer of the IRD. Sequence alignments were generated under the Hasegawa–Kishino–Yano (HKY) model [12] with the transition/transversion ratio being always estimated. The sequences were provides as unaligned FASTA.

## Results

### Development of the genotyping assay

To develop the genotypic assay using Sanger sequencing it was necessary to design universal primer pairs for each of influenza A and B virus genes which could generated RT-PCR product and also would be used in the dye terminator reaction. The universal primers earlier designed by Hoffmann et al. [10,11] amplify full-length genes of the viruses, which is not applicable if RNA is of poor quality. Since RNA concentration in NPW specimens is much less than in virus-containing allantoic fluid and most probably partially destroyed, some viral genes might be difficult to amplify in full length. Therefore, we designed additional primers annealing to conservative regions of viral genes which allowed amplification of their shorter regions (Table 2). For HA genes of H1N1 and H3N2 viruses a separate pair of primers were designed due to the high sequence variation. Sanger sequencing from the same universal primers can generate sequences of 300–500 nucleotides, which is sufficient to explicitly determine the origin of each viral gene.

**Table 2:** List of universal primers used in this study.

Type of influenza virus	Segment	Position	Sequence	Segment length, bp
A	PB2	F1757	TGG AAT TTG ARC CAT TT	~500
		R2250	AGA GTC CCG YTT TCG TTT CAT TAC	
	PB1	F594	AGT AAG RGA CAA CAT GAC CAA GAA	~500
		R1078	TGC CAT TTT RTT TGA GAA CAT TAT	
	PA	F1 [11]	TCA GGG AGC GAA AGC AGG TAC	~630
		R619	CTT CGC CTC TTT CGG ACT GAC G	
	HA/H1	F186	CGG GAA ACT ATG CAA ACT AAG AGG	~700
		R897	GCA ATC GTG GAC TGG TCT ATC TG	
	HA/H3	F234	AAA TAT GCG ACA GTC CTC A	~650
		R888	CAT TAT TGA GCT TTT CCC ACT TC	
	NP	F1 [11]	TCA GGG AGC AAA AGC AGG GTA	~730
		R715	TTG CAC ATT CTY TCA TAA GC	
	NA	F937	GTS TGC AGR GAY AAC TGG	~570
		R1500	TTA GTA GAA ACA AGG AGT TTT TT	
	M	F262	TGT CCA AAA TGC CCT MAA T	~770
		R1028	ATT AGT AGA AAC AAG GTA GTT TTT	
	NS	F1 [11]	TCA GGG AGC AAA AGC AGG GTG	~570
		R559	ATT GCA TTT TTG ACA TCC T	
B	PB2	F685	CAG TGG CAG GAG CAA CAT CA	~550
		R1233	CCT AGT GTC TTG AGA AAA TAC CAT	
	PB1	F353	TGG ARG CMC TAA TGG TCA CAA CTG	~500
		R831	ACC ACT TTG TTC TAG ATT TTC AC	
	PA	F318	CAA GAG CAT GGA ATA GAG ACT	~480
		R786	TAA CTG ATA CTA AGG GAG ACA T	
	HA	F1150	GAA GGA GGA TGG GAA GGA ATG AT	~410
		R1558	TCT GGT TGC ATT TGT GTT TGG TTT	
	NP	F1229	GGA GCT GCC TAT GAA GAC CTR AGA	~350
		R1557 [10]	ATA TCG TCT CGT ATT AGT AGA AAC AAC AGC ATT TTT TAC	
	NA	F1 [10]	TAT TCG TCT CAG GGA GCA GAA GCA GAG CA	~510
		R500	CTC TTG TTC CAT TGT AGT ATC C	
	M	F1 [10]	TAT TCG TCT CAG GGA GCA GAA GCA CGC ACT TTC TTA AAA TG	~600
		R583	CAT AGC TGA RAC CAT CTG C	
	NS	F490	TRA GGG ACA TGA ACA ACA AAG	~550
		R1100 [10]	ATA TCG TCT CGT ATT AGT AGT AAC AAG AGG ATT TTT AT	

**Fig 1:**
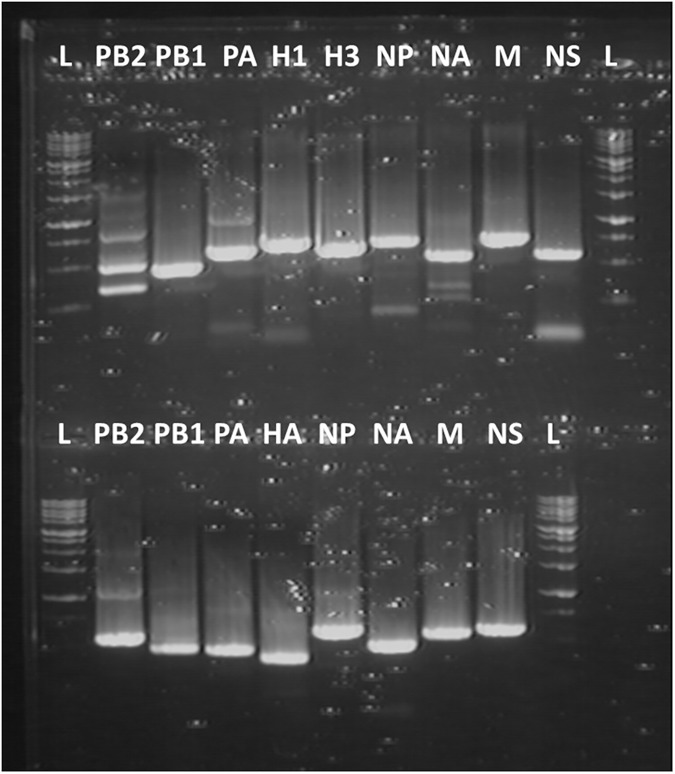
RT-PCR results with the trivalent LAIV using designed universal primers. The upper panel: influenza A-specific primers. The lower panel: influenza B-specific primers. L–DNA ladder.

We first tested the designed primers with RNA extracted from the trivalent LAIV containing influenza A(H1N1), A(H3N2) and B viruses. The result of RT-PCR is shown on Fig 1. All genes of type A and B components were amplified with high efficiency. The cDNAs were extracted from the gel and subjected to Sanger sequencing. The resulted nucleotide sequences showed 100% match with the corresponding sequences of the reassortant viruses contained in the vaccine (data not shown).

### Specificity of the genotyping assay

**Fig 2:**
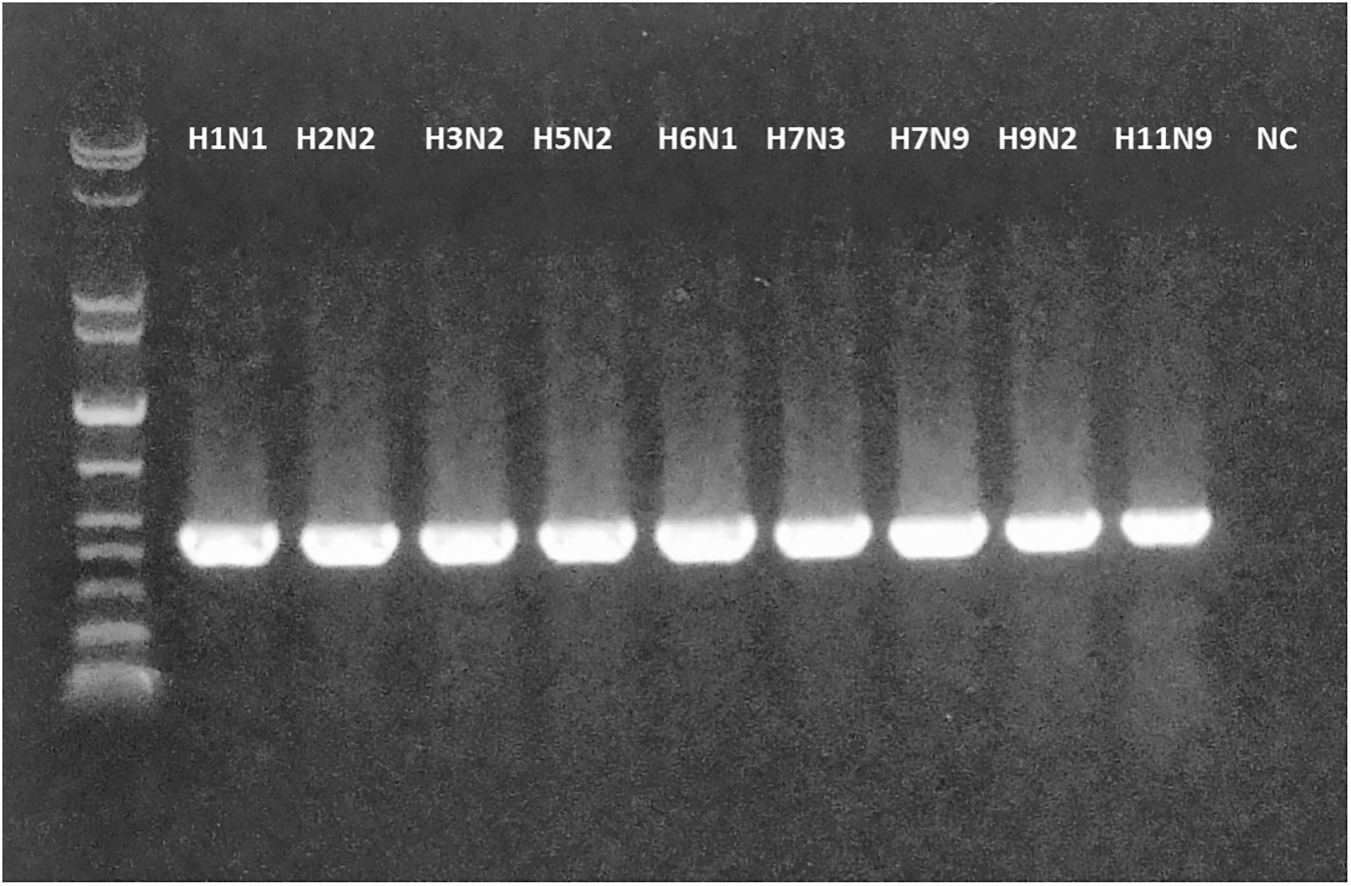
RT-PCR results of PB1 gene fragment of wild-type influenza A viruses of different subtypes using the newly designed universal primers. The strain names can be found in Table 1. NC, negative control.

We evaluated the feasibility of new assay to genotype a number of evolutionary unrelated influenza A and B viruses, to prove the versatility of the designed primers. In this case only primers for internal protein gene segments were tested, due to high sequence variation within the HA and NA genes of these viruses. Strikingly, the designed primers allowed amplifying genes of wild-type influenza A viruses of different subtypes, such as H1N1, H2N2, H3N2, H5N2, H6N1, H7N3, H7N9, H9N2, H11N9, as well as influenza B viruses of both Victoria and Yamagata lineages. The representative RT-PCR results for PB1 gene of influenza A viruses are shown on Fig 2. The extracted cDNA fragments were subjected to Sanger sequencing and the results uniquely determined the identity of each gene of the corresponding viruses, indicating the specificity of the assay (see S1 Fig for representative sequencing diagrams).

In addition, a BLAST search with each newly designed primer for internal proteins using the tools of Influenza Research Database (www.fludb.org) results in 100% homology match in at least 1000 influenza viruses of different subtypes, isolation years and species isolated, suggesting that the primers are indeed universal.

Since the new assay will be used for genotyping specimens derived from human subjects it was important to identify non-specific PCR products that could be potentially generated by annealing the designed primers to genomic DNA of human host. Although there are a number of human-specific PCR products that could be potentially generated from the designed primers, their length is different form the influenza-specific RT-PCR products (S2 Table). Our genotyping assay involves the extraction of viral cDNAs from agarose gels, therefore the potential human-origin DNA bands will not affect the assay outcome.

### Sensitivity of the genotyping assay

Sensitivity of the assay was studied using 10-fold dilutions of allantoic fluid containing 9.0 lgEID_50_/ml of A/Leningrad/134/17/57 or 9.2 lgEID_50_/ml of B/USSR/60/69 master donor viruses followed by viral RNA extraction from 140 μl of each dilution. The RT-PCR reactions were set up for each virus dilution in triplicates and the limit of detection (LoD) for each primer pair was determined as the minimal dose of virus capable of producing RT-PCR product which could be subsequently sequenced. The LoD was determined to be a virus concentration of 10^0.67∼2.67^ EID_50_/ml for Len/17 virus and 10^0.53∼2.2^ EID_50_/ml for B/USSR/60/69 virus (Table 3). This correlates to 10^−1.68∼0.46^ EID per reaction (2.6 μl/reaction), indicating high sensitivity of the genotyping assay.

**Table 3:** Limit of detection of influenza viral genes by new genotyping assay.

Gene segment	Limit of detection^†^, EID_50_/ml	
	A/Leningrad/134/17/57	B/USSR/60/69
PB2	10^1.33^	10^0.87^
PB1	10^0.67^	10^0.87^
PA	10^1.67^	10^0.53^
HA	n.d.^‡^	10^1.2^
NP	10^2.67^	10^1.87^
NA	10^0.67^	10^2.2^
M	10^0.67^	10^1.53^
NS	10^1.67^	10^1.53^

^†^Limit of detection is defined as the minimal dose of virus required to successfully amplify viral gene from newly designed primers

^‡^HA-specific primers were designed for H1N1 and H3N2 viruses only.

### Genotyping of the NPW specimens

#### Genotyping of the NPW specimens from phase II LAIV safety and immunogenicity clinical trial

The Phase II double-blind placebo-controlled clinical trials in Bangladesh was conducted to assess safety and immunogenicity of trivalent LAIV based on Len/17 backbone [13]. LAIV viral shedding was one of the outcomes measured during this trial; NPW specimens were collected from all study participants on days 2, 4 and 7 post-vaccination. Of the 300 study participants (150 in LAIV group and 150 in placebo group), 121 children were tested influenza-positive by ICDDR,B using conventional quantitative real-time RT-PCR protocol (qRT-PCR) [14] yielding total 287 positive NPW specimens. Additionally, influenza A-positive specimens were tested for the presence of H1, H3 or H5 subtype hemagglutinin. We employed our genotyping assay to determine if the positive specimens were wild-type or vaccine-type, and the specimens were masked during the study.

**Fig 3:**
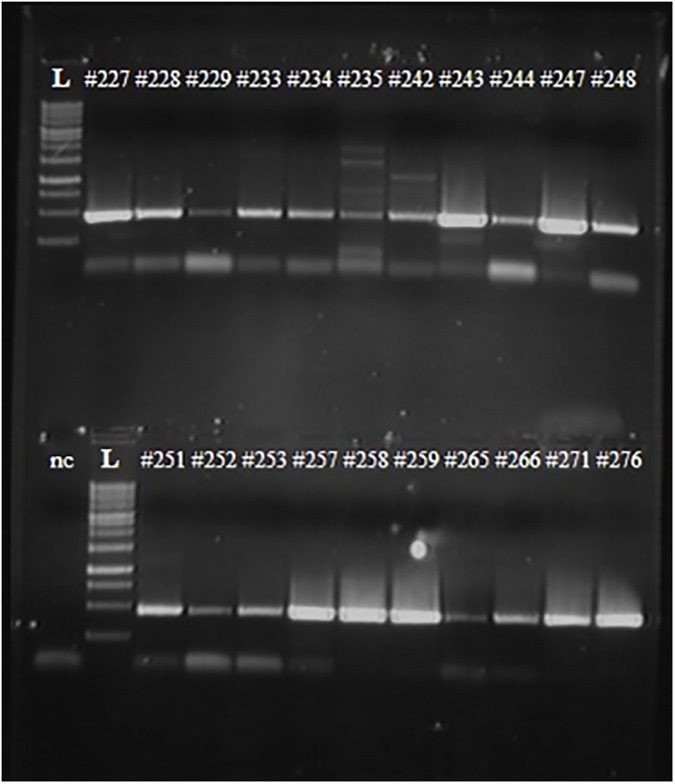
RT-PCR results of NPW samples with primers specific for PB1 gene of influenza A viruses. Numbers represent NPW specimens tested. L–DNA ladder. nc–negative control (no RNA added to the RT-PCR mixture).

As expected, the quality of the RNAs extracted from the samples was much poorer than the RNA extracted from virus-containing allantoic fluid. As a result, the quality of RT-PCR products (i.e. the intensity of the bands in agarose gel) significantly varied among the samples tested and the amount of extracted cDNA ranged from 20 ng to 400 ng, as measured by Qubit 3.0 fluorimeter (see representative Fig 3). Nevertheless, even cDNA extracted from weak bands could be successfully sequenced to conclude if the gene is vaccine-type or wild-type.

In general, if we could sequence one or both surface protein genes and one or more internal protein gene segments of a sample the results were considered conclusive, i.e. the origin of the virus (vaccine-type or wild-type) was determined. In case the sequencing results could be obtained for only surface genes, the results were considered inconclusive, as the HA and NA genes of circulating viruses might match the ones of LAIV strains. In addition, if sequencing results were obtained for internal protein genes of influenza A positive specimens, but neither HA nor NA could be amplified, these specimens were considered as unsubtyped influenza A viruses. Some representative sequencing diagrams generated during the study are given in Supplement (S2 and S3 Figs).

Table 4 shows the results of the genotyping of the NPW specimens after being unmasked. As expected, the vast majority of the isolates were determined to be vaccine-type, predominantly type A(H3N2) and type B LAIV components. No vaccine H1N1pdm strain could be detected in any of the NPW samples, however there were several specimens for which only internal protein gene segments could be sequenced and determined to be vaccine-type. Nevertheless, qRT-PCR data for these specimens suggest that they were also of H3N2 subtype. Importantly, seven qRT-PCR influenza-positive NPW samples were detected in placebo group, highlighting the importance of virus genotyping, as high level of LAIV virus transmission to the unvaccinated contacts would be suspected. All but one of them were genotyped as wild-type influenza infection: 3 subjects (or 6 NPW samples collected at different time points) shed wild-type influenza B virus of Yamagata lineage (Table 4). There was a single NPW specimen in the placebo group which was tested positive for vaccine-type influenza B virus, indicating either the vaccine virus transmission or possible sample mishandling. Of note, a single transmission event described for the Ann Arbor-backbone LAIV also occurred in a child with type B LAIV component [4].

**Table 4:** Summary of testing of NPW specimens collected during phase II LAIV trial in Bangladesh (samples collected on days 2, 4 and 7 after vaccination).

Study group	Type	Subtype/ lineage	NPW specimens by qRT-PCR (n = 287)		NPW specimens by sequencing (n = 284^3^)			
			N positive^1^	Ct value range	N positive^4^	Vaccine-type	Wild-type	Inconclusive
LAIV	A	H1N1pdm	1	38.25	0	-	-	-
		H3N2	138	21.90–39.96	134	133	0	1
		Unsubtyped	0	-	6^5^	6	0	0
	B	Yamagata	196^2^	20.27–41.18	0	-	-	-
		Victoria			223	221	1	1
Placebo	A	H1N1pdm	0	-	0	-	-	-
		H3N2	0	-	0	-	-	-
		Unsubtyped	0	-	0	-	-	-
	B	Yamagata	7^2^	20.90–38.86	6	0	6	0
		Victoria			1	1	0	0

^1^co-infection with two viruses was detected in 55 subjects

^2^qRT-PCR protocol did not distinguish influenza B lineages

^3^Three NPW specimens positive by qRT- PCR (Ct values 34.50–42.66) were negative by RT-PCR

^4^co-infection with two or three viruses was detected in 86 subjects

^5^positive for H3/HA by qRT-PCR (Ct values 35.71–39.96).

Three of the 287 NPW samples positive by qRT-PCR could not be amplified under our conditions, even after several attempts with additional RNA extraction steps. We conclude that the viral RNA in these samples has been destroyed during the storage of the samples and it is impossible to discern which were vaccine-type and which were wild-type. Overall, our assay showed higher sensitivity than qRT-PCR assay since we were able to genotype totally 370 viruses from the 287 NPW samples, whereas qRT-PCR detected only 342 viruses (Table 4). These data indicate that the use of a single qRT-PCR assay to detect influenza virus in LAIV-immunized children might significantly under-estimate the level of LAIV virus shedding. Noteworthy, the efficacy of virus genotyping by new assay did not correlate with either the qRT-PCR Ct values or the concentration of RNA extracted from the NPW specimens.

#### Genotyping of the NPW specimens from phase III LAIV clinical efficacy trial in Bangladesh

The second set of influenza-positive NPW specimens was collected from children immunized with trivalent LAIV or placebo that met protocol-defined criteria of illness within 14 days of vaccination [15]. Differentiation of wild-type and vaccine-type influenza viruses in the positive NPW specimens was critical to assess the safety of the LAIV because vaccination took place during active circulation of influenza viruses in the community [15]. Sixty nine NPW samples from symptomatic cases were tested positive by conventional WHO qRT-PCR protocol [14], 19 of which resulted from co-infection of at least two different influenza viruses (Table 5). Importantly, all influenza-positive specimens obtained from placebo group were identified as wild-type H1N1pdm viruses. In addition, 12 children in the LAIV group were infected with these viruses within two weeks of vaccination resulting in mixed LAIV-wild-type viral infections in at least six subjects. Strikingly, no mixtures were seen on sequencing diagrams of viral internal protein genes in subjects co-infected with type A LAIV and H1N1pdm wild-type virus, presumably due to the prevalence of one viral population over another. The minor component could be detected only by amplification of viral HA gene. Overall, the majority of the NPW specimens were genotyped as vaccine-type: 40 subjects were positive for LAIV type A virus and 36 participants were positive for LAIV type B virus (Table 5). Only two qRT-PCR-positive NPW specimens could not be amplified under our conditions.

**Table 5:** Summary of testing of NPW specimens collected during phase III LAIV trial in Bangladesh (samples collected from symptomatic cases within 2 weeks of vaccination).

Study group	Type	Subtype/ lineage	NPW specimens by real-time RT-PCR (n = 69)		NPW specimens by sequencing (n = 67^3^)			
			N positive^1^	Ct value range	N positive^4^	Vaccine-type	Wild-type	Inconclusive
LAIV	A	H1N1pdm	11	18.17–34.08	12	0	12	0
		H3N2	36	21.56–38.85	37	37	0	0
		Unsubtyped	3	35.87–38.18	2	2	0	0
	B	Yamagata	29^2^	25.26–39.75	36	36	0	0
		Victoria			0	-	-	-
Placebo	A	H1N1pdm	9	19.72–27.66	9	0	9	0
		H3N2	0	-	0	-	-	-
		Unsubtyped	0	-	0	-	-	-
	B	Yamagata	0	-	0	-	-	-
		Victoria			0	-	-	-

^1^co-infection with two viruses was detected in 19 subjects

^2^rRT-PCR protocol did not distinguish influenza B lineages

^3^Two NPW specimens positive by real-time PCR (Ct values 37.58, 35.7) were negative by RT-PCR

^4^co-infection of two or three viruses was detected in 23 subjects

#### Analysis of wild-type influenza viruses detected in NPW specimens

**Fig 4:**
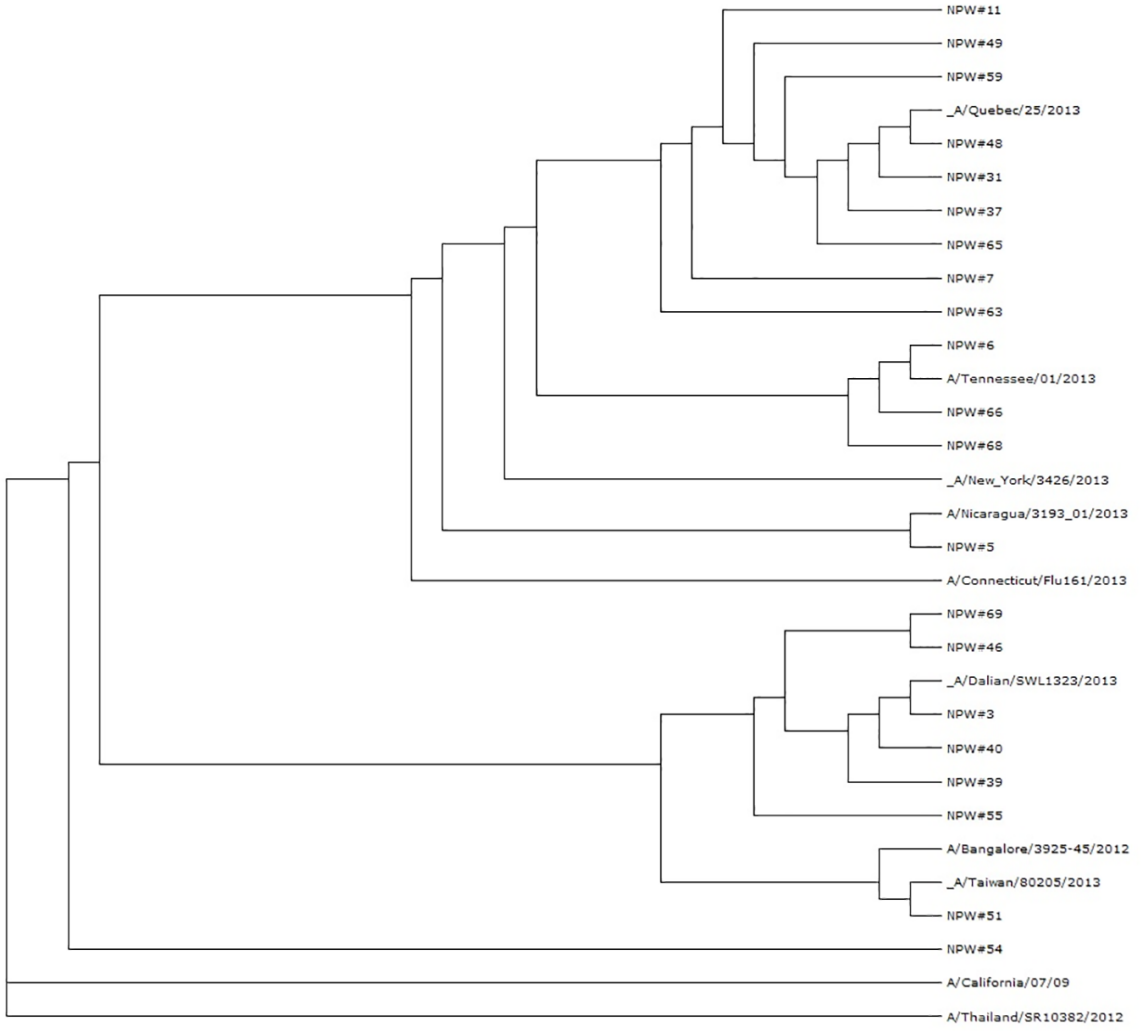
Evolutionary relationship between HAs of wild-type H1N1 viruses detected in NPW samples of phase III LAIV trial in Bangladesh. The tree was generated using Archaeopteryx Phylogenetic Tree Viewer of the Influenza Research Database (fludb.org).

The new genotyping assay allows analyzing genetic features of wild-type influenza viruses which circulated during the LAIV trial. Interestingly, the detected wild-type H1N1pdm influenza viruses differed significantly among subject, and even short regions of their genes sequenced by new assay were sufficient to follow their evolutionary relationship (Fig 4). Twelve of 22 (54.5%) isolates matched with the HA fragment of A/New York/3426/2013 virus; whereas the remaining 10 viruses had unique combinations of nucleotide substitutions within this sequenced HA fragment. In addition, the circulating type B influenza viruses identified during the trial belonged to both lineages, Victoria and Yamagata. Thus, the identification of up to 500 nucleotides for each gene of the wild-type virus gives an opportunity to find evolutionary relationship with the other influenza viruses deposited in the GenBank.

Altogether, the new genotyping assay is sensitive and specific approach capable of identifying the origin of influenza virus infection in LAIV-immunized individuals.

## Discussion

Various assays have been previously developed to distinguish wild-type and vaccine-type viruses during the process of LAIV reassortant strain preparation, also known as genome composition analysis [16–20]. Of them only “partial sequencing” strategy could be employed to differentiate LAIV virus from unknown wild-type virus in a field clinical trials because this assay is based on the amplification of viral gene (full or in part) from primers annealing to very conservative gene regions (i.e. universal primers) [19]. The identification of nucleotide sequences of these gene regions (300–500 nucleotides) from the same universal primer can explicitly determine gene origin. Here we report the designing of new universal primers annealing to the conservative regions within all influenza A and B viral genes. These primers, in some cases used in pairs with previously designed universal primers [10,11], allow amplification of shorter gene fragments, therefore increasing the sensitivity of the assay. Importantly, we were able to demonstrate the specificity of the assay using influenza A viruses of various subtypes of human and avian origins, as well as both lineages of influenza B virus. These findings indicate that this assay can be successfully used to genotype virtually any influenza-positive NPW specimen, with possibility of finding natural genetic reassortants in the field isolates.

The new assay was successfully applied for the genotyping of influenza-positive NPW specimens obtained during clinical trials of seasonal trivalent Russian-backbone LAIV conducted in Bangladesh, a tropical country without distinct pattern of influenza seasonality. Viral RNA extracted from the NPW specimens is most probably of not a very good quality since the LAIV viruses do not replicate to high titers in the upper respiratory tract of vaccinees [21]. For this reason it was unlikely to amplify viral genome segments in full length using universal primers complementary to their conservative non-coding regions [10,11,22]. Indeed, the intensity of the cDNA bands of even short gene regions varied significantly among the NPW specimens. Nevertheless, Sanger sequencing of the extracted cDNAs gave comprehensive information on their origins.

We identified that the most of the positive NPW specimens originated from LAIV viruses, however a number of wild-type influenza A(H1N1) and B viruses have been identified. The detection of vaccine viruses in children seeking medical care for influenza-like illness (ILI) for the first week of vaccination is not surprising. It is known that LAIV can cause mild ILI symptoms upon vaccination, and even low levels of moderate reactions are acceptable by regulatory agencies [2,23]. Children presented with ILI during the first week of vaccination in the Phase III efficacy trial mostly had mild symptoms, and therefore the cause of these symptoms could be the vaccine [9]. Importantly, genotyping of influenza-positive NPW samples in the placebo group as wild-type influenza viruses allowed avoiding the conclusion that LAIV is easily transmissible between children. In contrast, only a single positive specimen from the control group was genotyped as LAIV type B virus, suggesting very low probability of the vaccine virus transmission between close contacts. Interestingly, the study by Block et al. [3] identified influenza-positive cultures in several subjects immunized with Ann Arbor-based LAIV as late as 9–28 days after vaccination, which were most probably false positives. Unfortunately, the authors could not determine the origin of these positive specimens, and if they had used a genotyping assay similar to the one described here, they would have shed light on whether the LAIV viruses are capable of this prolonged replication.

We were unable to detect H1N1 LAIV component in any of the NPW specimen, which is consistent with previous findings of the lack of 2009 H1N1 pandemic LAIV shedding in both children and adult age groups [24–26]. Nevertheless, these vaccines were efficacious in preventing pandemic influenza infection in both age groups [27,28]. In addition, a trivalent LAIV tested in the phase III trial in Bangladesh was also efficacious against H1N1pdm viruses [15].

Standard influenza A and influenza B qRT-PCR protocols are based on the detection of one short type/subtype-specific fragment of one of eight viral genes. The oligonucleotide primers and probes for detection of Influenza A, 2009 Influenza A (swine origin), and B viruses were selected from highly conserved regions of the matrix (M), nucleoprotein (NP), and non-structural (NS) genes, respectively (http://www.accessdata.fda.gov/cdrh_docs/reviews/K111507.pdf). These primers should be highly specific to a particular influenza type/subtype and cannot react with the other viruses, whereas our approach is based on the universal primers that amplify the vast majority of human and avian influenza viruses. Subsequent Sanger sequencing and comparison with known sequences deposited in GenBank allows identification of the source of the viral genes. It is not surprising that we were capable to detect more influenza viruses in the NPW specimens than were detected by qRT-PCR, since we tested at least four viral genes by the genotyping assay. This might not be relevant for the routine testing of NPW samples from patients with influenza-like illnesses due to the higher cost, however in specific studies with the need to closely monitor the origin of the virus isolates the new assay will be a valuable tool.

One of the possible improvements of the new genotyping assay is the use of pyrosequencing instead of Sanger sequencing. Standard pyrosequencing is a rapid method of generating accurate sequences up to 50 nucleotides, and has been used for the detection and subtyping of human influenza viruses and reassortants, both naturally-occurred and produced during the process of LAIV strain development [18,29]. However, our strategy allows sequencing of longer gene fragments giving an opportunity to track evolutionary relationship between circulating wild-type viruses, including the detection of possible genetic reassortant variants in nature.

A disadvantage of genotyping assays using Sanger sequencing technology is the limited power to detect minor viral quasispecies within a single sample. Although apparent co-infections were detected in some NPW specimens (i.e. both H1N1 wt and H3N2 LAIV viruses have been simultaneously identified), no mixed nucleotides were seen on sequencing chromatograms of internal protein genes. Recent advances in high throughput sequencing technologies have led to the opportunity to analyze many viral genomes in parallel in a single experiment, capable of detecting minor viral variants in genetically heterogeneous populations [30–32]. The majority of new generation sequencing approaches require input DNA of high quality, therefore if these new technologies are to be used for genotyping of influenza viruses directly from clinical samples such as NPW, the primers designed in our study might be useful to generate viral-specific cDNAs of satisfactory quality without interference with human genomic DNA present in the specimens.

## Supporting information

S1 Table Accession numbers of partial sequences of wild-type influenza virus genes generated during this study. (DOCX)

S2 Table Prediction of non-specific amplification of human genomic DNA using new influenza A and B universal primer set (PCR products less than 2000 bp are shown). (DOCX)

S1 Fig Sequencing chromatograms of PB1 gene of wild-type influenza A viruses. Upper sequences without peaks: PB1 gene fragments obtained from sequence database. Lower sequences with peaks: sequencing diagrams generated by Sanger sequencing using new PB1-specific universal primers.
(TIF)

S2 Fig Sequencing chromatograms of HA gene fragment of influenza A viruses using new H3-specific universal primers. H3_trivac–HA gene fragment of trivalent LAIV amplified with H3-specific primers.
(TIF)

S3 Fig Sequencing chromatograms of PB1 gene fragment of influenza A viruses using new universal primers. PB1_trivac–PB1 gene fragment of trivalent LAIV amplified with PB1-specific primers.
(TIF)
