# Supplementary material for: Genotyping assay for differentiation of wild-type and vaccine viruses in subjects immunized with live attenuated influenza vaccine
Source: PLoS One. 2017 Jul 7;12(7):e0180497. doi: 10.1371/journal.pone.0180497 (PMC5501548; doi:10.1371/journal.pone.0180497)
Supplement: S1 Table — (DOCX) [file pone.0180497.s001.docx]

**S1 Table.** Accession numbers of partial sequences of wild-type influenza virus genes generated during this study

| NPW Lab No | Virus type | Subtype/  lineage | GISAID Isolate ID | Gene segment | GISAID Accession No |
| --- | --- | --- | --- | --- | --- |
| **LAIV Phase II safety and immunogenicity clinical trial in Bangladesh** | | | | | |
| 91 | B | Yamagata | EPI_ISL_237510 | HA | EPI858658 |
|  |  |  |  | NA | EPI858692 |
|  |  |  |  | NS | EPI858693 |
| 246 | B | Victoria | EPI_ISL_237517 | PB1 | EPI858697 |
|  |  |  |  | PA | EPI858696 |
|  |  |  |  | NA | EPI858694 |
|  |  |  |  | NS | EPI858695 |
| 260 | B | Yamagata | EPI_ISL_237518 | HA | EPI858698 |
|  |  |  |  | NA | EPI858699 |
|  |  |  |  | NS | EPI858700 |
| 267 | B | Yamagata | EPI_ISL_237519 | HA | EPI858701 |
|  |  |  |  | NA | EPI858702 |
|  |  |  |  | NS | EPI858703 |
| **LAIV Phase III clinical efficacy trial in Bangladesh** | | | | | |
| 3 | A | H1N1pdm | EPI_ISL_237520 | PB1 | EPI858707 |
|  |  |  |  | HA | EPI858704 |
|  |  |  |  | NA | EPI858706 |
|  |  |  |  | M | EPI858705 |
| 5 | A | H1N1pdm | EPI_ISL_237521 | PB1 | EPI858711 |
|  |  |  |  | HA | EPI858708 |
|  |  |  |  | NA | EPI858710 |
|  |  |  |  | M | EPI858709 |
| 6 | A | H1N1pdm | EPI_ISL_237522 | PB2 | EPI858716 |
|  |  |  |  | PB1 | EPI858715 |
|  |  |  |  | HA | EPI858712 |
|  |  |  |  | NA | EPI858714 |
|  |  |  |  | M | EPI858713 |
| 7 | A | H1N1pdm | EPI_ISL_237523 | PB2 | EPI858717 |
|  |  |  |  | PB1 | EPI858718 |
|  |  |  |  | HA | EPI858721 |
|  |  |  |  | NA | EPI858719 |
|  |  |  |  | M | EPI858720 |
| 11 | A | H1N1pdm | EPI_ISL_237524 | HA | EPI858722 |
| 31 | A | H1N1pdm | EPI_ISL_237525 | PB1 | EPI858726 |
|  |  |  |  | HA | EPI858723 |
|  |  |  |  | NA | EPI858725 |
|  |  |  |  | M | EPI858724 |
| 37 | A | H1N1pdm | EPI_ISL_237526 | PB1 | EPI858727 |
|  |  |  |  | HA | EPI858730 |
|  |  |  |  | NA | EPI858728 |
|  |  |  |  | M | EPI858729 |
| 39 | A | H1N1pdm | EPI_ISL_237527 | PB1 | EPI858734 |
|  |  |  |  | HA | EPI858731 |
|  |  |  |  | NA | EPI858733 |
|  |  |  |  | M | EPI858732 |
| 40 | A | H1N1pdm | EPI_ISL_237528 | PB1 | EPI858735 |
|  |  |  |  | HA | EPI858738 |
|  |  |  |  | NA | EPI858736 |
|  |  |  |  | M | EPI858737 |
| 46 | A | H1N1pdm | EPI_ISL_237529 | PB1 | EPI858742 |
|  |  |  |  | HA | EPI858739 |
|  |  |  |  | NA | EPI858741 |
|  |  |  |  | M | EPI858740 |
| 48 | A | H1N1pdm | EPI_ISL_237530 | PB1 | EPI858743 |
|  |  |  |  | HA | EPI858746 |
|  |  |  |  | NA | EPI858744 |
|  |  |  |  | M | EPI858745 |
| 49 | A | H1N1pdm | EPI_ISL_237531 | PB1 | EPI858750 |
|  |  |  |  | HA | EPI858747 |
|  |  |  |  | NA | EPI858749 |
|  |  |  |  | M | EPI858748 |
| 51 | A | H1N1pdm | EPI_ISL_237532 | PB1 | EPI858751 |
|  |  |  |  | HA | EPI858754 |
|  |  |  |  | NA | EPI858752 |
|  |  |  |  | M | EPI858753 |
| 54 | A | H1N1pdm | EPI_ISL_237533 | PB1 | EPI858758 |
|  |  |  |  | HA | EPI858755 |
|  |  |  |  | NA | EPI858757 |
|  |  |  |  | M | EPI858756 |
| 55 | A | H1N1pdm | EPI_ISL_237534 | PB1 | EPI858759 |
|  |  |  |  | HA | EPI858762 |
|  |  |  |  | NA | EPI858760 |
|  |  |  |  | M | EPI858761 |
| 59 | A | H1N1pdm | EPI_ISL_237535 | PB1 | EPI858766 |
|  |  |  |  | HA | EPI858763 |
|  |  |  |  | NA | EPI858765 |
|  |  |  |  | M | EPI858764 |
| 63 | A | H1N1pdm | EPI_ISL_237536 | PB1 | EPI858767 |
|  |  |  |  | HA | EPI858770 |
|  |  |  |  | NA | EPI858768 |
|  |  |  |  | M | EPI858769 |
| 65 | A | H1N1pdm | EPI_ISL_237537 | PB1 | EPI858774 |
|  |  |  |  | HA | EPI858771 |
|  |  |  |  | NA | EPI858773 |
|  |  |  |  | M | EPI858772 |
| 66 | A | H1N1pdm | EPI_ISL_237538 | PB1 | EPI858775 |
|  |  |  |  | HA | EPI858778 |
|  |  |  |  | NA | EPI858776 |
|  |  |  |  | M | EPI858777 |
| 68 | A | H1N1pdm | EPI_ISL_237539 | PB1 | EPI858781 |
|  |  |  |  | HA | EPI858777 |
|  |  |  |  | M | EPI858780 |
| 69 | A | H1N1pdm | EPI_ISL_237540 | PB1 | EPI858782 |
|  |  |  |  | HA | EPI858785 |
|  |  |  |  | NA | EPI858783 |
|  |  |  |  | M | EPI858784 |
