# Supplementary material for: Genotyping assay for differentiation of wild-type and vaccine viruses in subjects immunized with live attenuated influenza vaccine
Source: PLoS One. 2017 Jul 7;12(7):e0180497. doi: 10.1371/journal.pone.0180497 (PMC5501548; doi:10.1371/journal.pone.0180497)
Supplement: S2 Table — (DOCX) [file pone.0180497.s002.docx]

**S2 Table.** Prediction of non-specific amplification of human genomic DNA using new influenza A and B universal primer set (PCR products less than 2000 bp are shown)

| Virus type | Viral gene | Expected size for viral RT-PCR product | Predicted annealing to human genomic DNA  (homo sapiens, GRCh38.p7) ^†^ | | | | |
| --- | --- | --- | --- | --- | --- | --- | --- |
|  |  |  | Chromosome # | Accession number | Product length, bp. | Primer pair^‡^ | Primer homology |
| A | PB2 | 500 | - | - | - | - | - |
|  | PB1 | 500 | 20 | [NC_000020.11](https://www.ncbi.nlm.nih.gov/nucleotide/568815578?from=12944137&to=12944349&report=gbwithparts) | 213 | FR | 81% |
|  |  |  | 4 | [NC_000004.12](https://www.ncbi.nlm.nih.gov/nucleotide/568815594?from=90012781&to=90012999&report=gbwithparts) | 219 | FR | 79% |
|  |  |  | 9 | [NC_000009.12](https://www.ncbi.nlm.nih.gov/nucleotide/568815589?from=137941916&to=137942133&report=gbwithparts) | 218 | FR | 79% |
|  | PA | 630 | 22 | [NC_000022.11](https://www.ncbi.nlm.nih.gov/nucleotide/568815576?from=37322969&to=37323006&report=gbwithparts) | 38 | FF | 76% |
|  | H1 | 700 | - | - | - | - | - |
|  | H3 | 650 | 6 | [NC_000006.12](https://www.ncbi.nlm.nih.gov/nucleotide/568815592?from=139709349&to=139710116&report=gbwithparts) | 768 | RR | 83% |
|  | NP | 730 | 8 | [NC_000008.11](https://www.ncbi.nlm.nih.gov/nucleotide/568815590?from=96658129&to=96658730&report=gbwithparts) | 602 | FR | 83% |
|  |  |  | 1 | [NC_000001.11](https://www.ncbi.nlm.nih.gov/nucleotide/568815597?from=107787304&to=232940275&report=gbwithparts) | 636 | FF | 81% |
|  |  |  | 4 | [NC_000004.12](https://www.ncbi.nlm.nih.gov/nucleotide/568815594?from=66929689&to=66931582&report=gbwithparts) | 1894 | RR | 75% |
|  |  |  | 11 | [NC_000011.10](https://www.ncbi.nlm.nih.gov/nucleotide/568815587?from=58247765&to=58249538&report=gbwithparts) | 1774 | RR | 75% |
|  | NA | 570 | 21 | [NC_000021](https://www.ncbi.nlm.nih.gov/nuccore/NC_000021) | 359 | FF | 83% |
|  |  |  | X | [NC_000023.11](https://www.ncbi.nlm.nih.gov/nucleotide/568815575?from=80565237&to=80566542&report=gbwithparts) | 1306 | FF | 80% |
|  |  |  | 6 | [NC_000006.12](https://www.ncbi.nlm.nih.gov/nucleotide/568815592?from=45083866&to=45083940&report=gbwithparts) | 75 | FF | 78% |
|  |  |  | 11 | [NC_000011.10](https://www.ncbi.nlm.nih.gov/nucleotide/568815587?from=5126368&to=18951226&report=gbwithparts) | 86 | FF | 78% |
|  |  |  | 7 | [NC_000007.14](https://www.ncbi.nlm.nih.gov/nucleotide/568815591?from=78883776&to=78884853&report=gbwithparts) | 1078 | FR | 78% |
|  | M | 770 | - | - | - | - | - |
|  | NS | 570 | 8 | [NC_000008.11](https://www.ncbi.nlm.nih.gov/nucleotide/568815590?from=62904321&to=80052206&report=gbwithparts) | 370 | FR | 85% |
|  |  |  |  |  | 560 | FR | 80% |
|  |  |  | 4 | [NC_000004.12](https://www.ncbi.nlm.nih.gov/nucleotide/568815594?from=58819667&to=58820566&report=gbwithparts) | 900 | RR | 79% |
|  |  |  | 5 | [NC_000005.10](https://www.ncbi.nlm.nih.gov/nucleotide/568815593?from=1286368&to=1287883&report=gbwithparts) | 1516 | FR | 80% |
|  |  |  | 12 | [NC_000012.12](https://www.ncbi.nlm.nih.gov/nucleotide/568815586?from=7527318&to=7528143&report=gbwithparts) | 826 | FF | 79% |
|  |  |  | 14 | [NC_000014.9](https://www.ncbi.nlm.nih.gov/nucleotide/568815584?from=98728029&to=98728159&report=gbwithparts) | 131 | RR | 76% |
| B | PB2 | 550 | 4 | [NC_000004.12](https://www.ncbi.nlm.nih.gov/nucleotide/568815594?from=172897419&to=172897851&report=gbwithparts) | 433 | FF | 80% |
|  | PB1 | 500 | 9 | [NC_000009.12](https://www.ncbi.nlm.nih.gov/nucleotide/568815589?from=16948902&to=16949545&report=gbwithparts) | 644 | RR | 78% |
|  | PA | 480 | 10 | [NC_000010.11](https://www.ncbi.nlm.nih.gov/nucleotide/568815588?from=18560726&to=18565970&report=gbwithparts) | 1027 | FF | 79% |
|  |  |  |  |  | 1670 | FF | 79% |
|  |  |  |  |  | 1849 | FF | 79% |
|  |  |  |  |  | 1946 | FF | 79% |
|  |  |  | 13 | [NC_000013.11](https://www.ncbi.nlm.nih.gov/nucleotide/568815585?from=108629692&to=108630316&report=gbwithparts) | 625 | FF | 81% |
|  |  |  | 8 | [NC_000008.11](https://www.ncbi.nlm.nih.gov/nucleotide/568815590?from=124061645&to=124062385&report=gbwithparts) | 741 | FR | 77% |
|  | HA | 410 | 3 | [NC_000003.12](https://www.ncbi.nlm.nih.gov/nucleotide/568815595?from=72624826&to=72626688&report=gbwithparts) | 1863 | FR | 79% |
|  |  |  |  |  | 1811 | FR | 79% |
|  | NP | 350 | - | - | - | - | - |
|  | NA | 510 | - | - | - | - | - |
|  | M | 600 | 16 | [NC_000016.10](https://www.ncbi.nlm.nih.gov/nucleotide/568815582?from=29110837&to=87980514&report=gbwithparts) | 1703 | RR | 89% |
|  |  |  |  |  | 1703 | RR | 89% |
|  | NS | 550 | 15 | [NC_000015.10](https://www.ncbi.nlm.nih.gov/nucleotide/568815583?from=73896074&to=73897789&report=gbwithparts) | 1716 | FF | 83% |

^†^ analysis was performed using Genome Reference Consortium Human Build 38 patch release 7 (GRCh38.p7), GenBank assembly accession: GCA_000001405.22 (replaced);

^‡^ F: forward primer; R: reverse primer. A single primer can anneal to both DNA strands and generate a PCR product.
