# Supplementary figures and images for: Genotyping assay for differentiation of wild-type and vaccine viruses in subjects immunized with live attenuated influenza vaccine
Source: PLoS One. 2017 Jul 7;12(7):e0180497. doi: 10.1371/journal.pone.0180497 (PMC5501548; doi:10.1371/journal.pone.0180497)

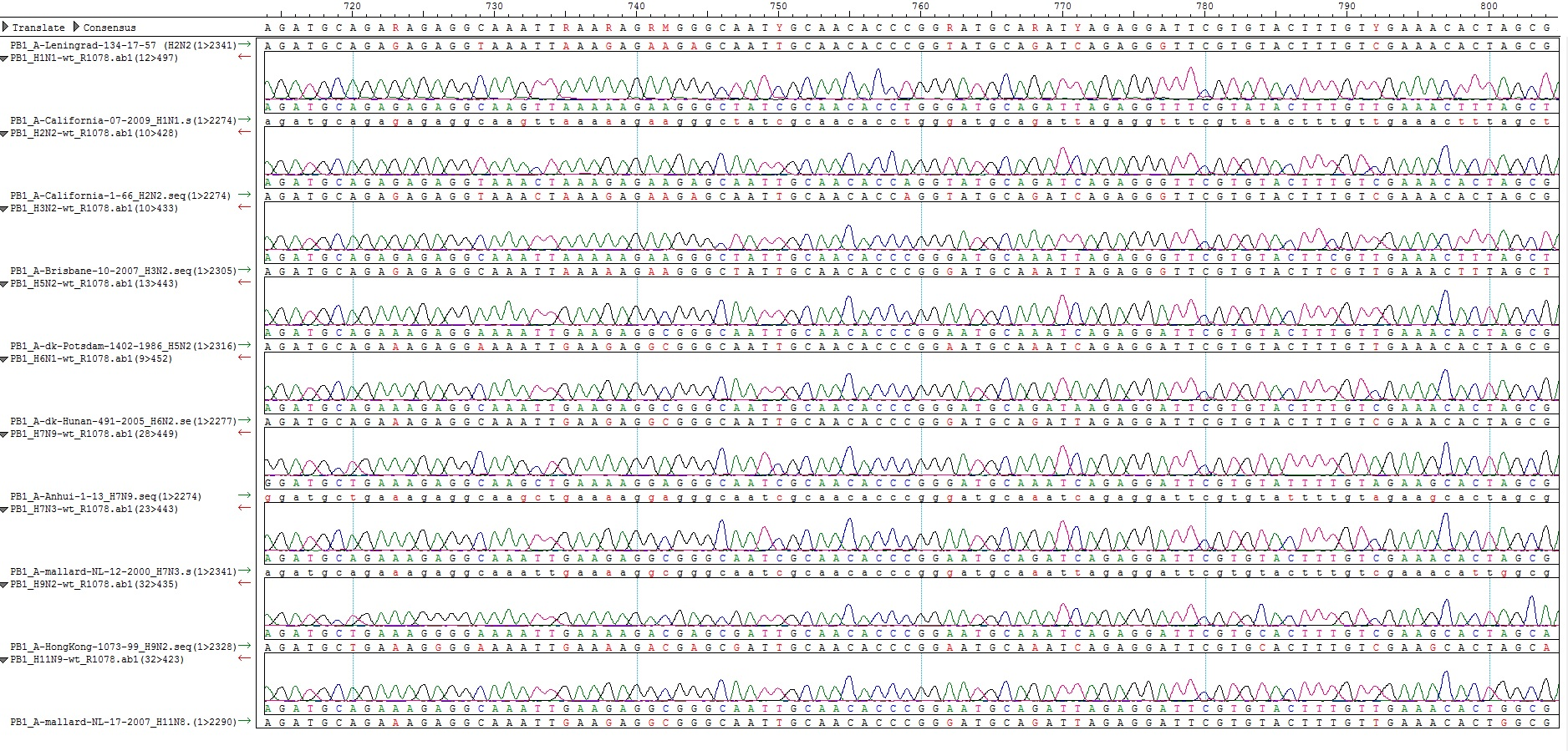

Supplement: S1 Fig — Upper sequences without peaks: PB1 gene fragments obtained from sequence database. Lower sequences with peaks: sequencing diagrams generated by Sanger sequencing using new PB1-specific universal primers. (TIF) [file pone.0180497.s003.tif]

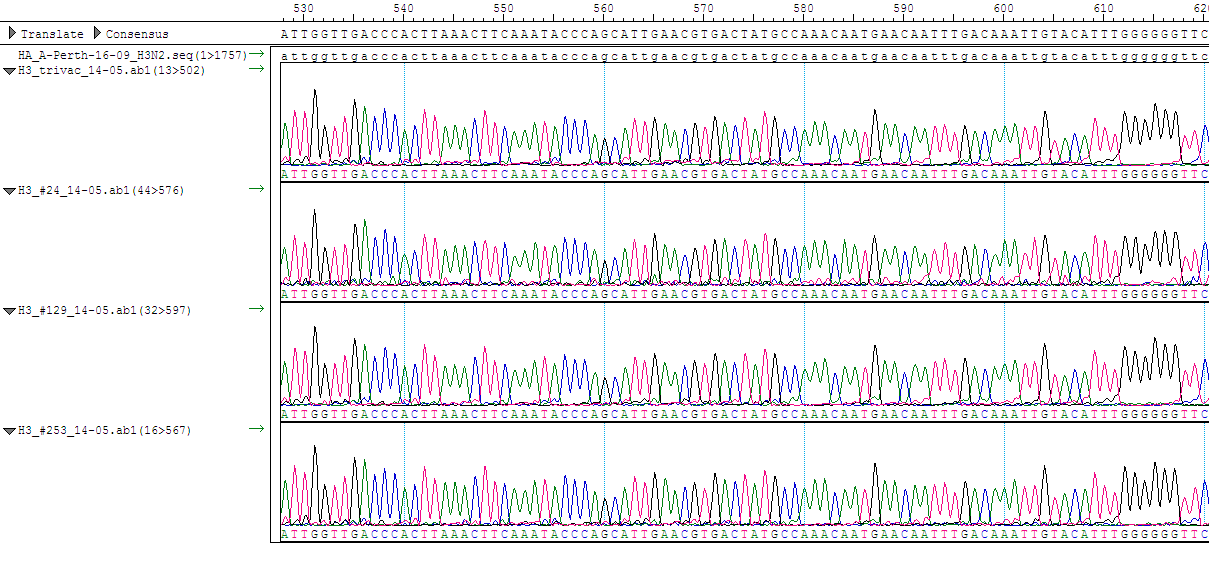

Supplement: S2 Fig — H3_trivac–HA gene fragment of trivalent LAIV amplified with H3-specific primers. (TIF) [file pone.0180497.s004.tif]

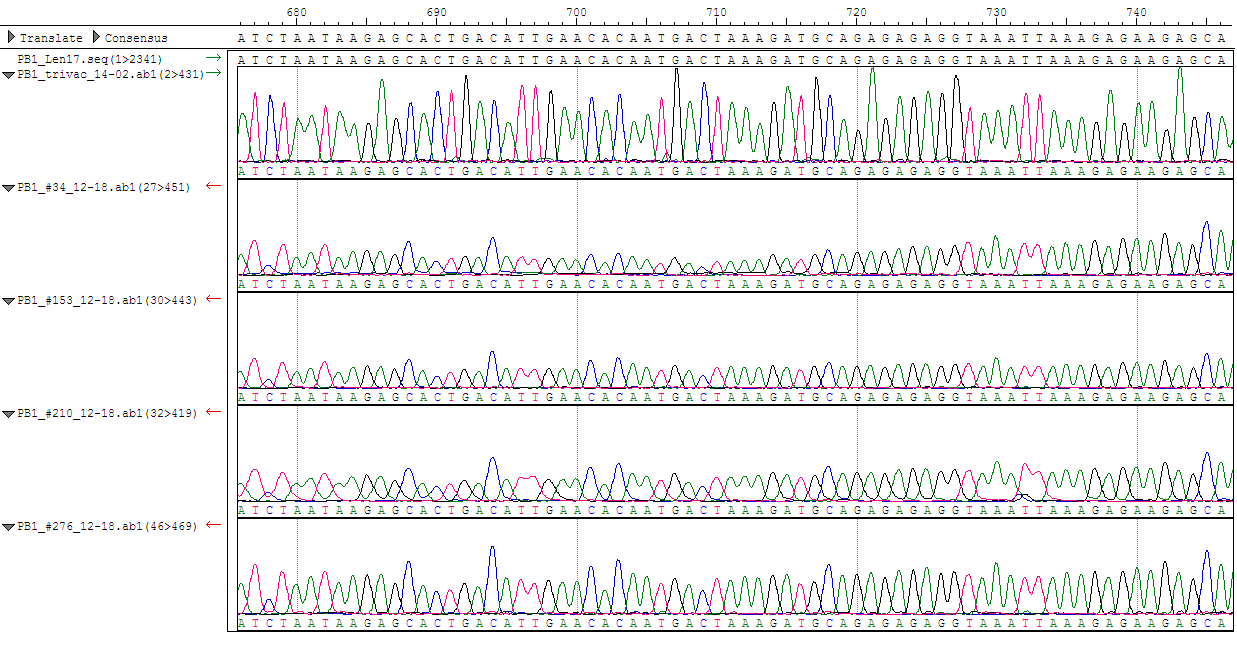

Supplement: S3 Fig — PB1_trivac–PB1 gene fragment of trivalent LAIV amplified with PB1-specific primers. (TIF) [file pone.0180497.s005.tif]
